# Enhanced Morphology-Dependent Tensile Property and Breakdown Strength of Impact Polypropylene Copolymer for Cable Insulation

**DOI:** 10.3390/ma13183935

**Published:** 2020-09-05

**Authors:** Kai Yang, Yun Liu, Zhimin Yan, Ye Tian, Yitao Liu, Zhenghong Jing, Jianying Li, Shengtao Li

**Affiliations:** 1 State Key Laboratory of Electrical Insulation and Power Equipment, Xi’an Jiaotong University, Xi’an 710049, China; yk237564129@stu.xjtu.edu.cn (K.Y.); ly250xjtu@stu.xjtu.edu.cn (Y.L.); yanzhimin@stu.xjtu.edu.cn (Z.Y.); sli@mail.xjtu.edu.cn (S.L.); 2 Electric Power Research Institute of Liaoning Power Grid Company Limited, Shenyang 110000, China; tianyemailbox@126.com (Y.T.); Liuyitao-71@sohu.com (Y.L.); 3 Sinopec Yanshan Petrochemical Company, Beijing 102500, China; jingzh.yssh@sinopec.com

**Keywords:** impact polypropylene copolymer, elongation at break, breakdown strength, morphology, rubber microspheres, crystalline structure, trap distribution

## Abstract

The decrease in electrical properties caused by the toughening of polypropylene (PP) is a difficult problem for the modification of PP used for cable insulation. In this research, an isotactic PP, a cross-linked polyethylene (XLPE) and two impact PP copolymers (IPCs) with an ethylene–propylene rubber phase content of 15 and 30% were prepared to assess the possibility of IPCs to be used as cable insulating material. The tensile properties and breakdown strength were evaluated, meanwhile, the rubber phase content dependence of the crystalline structure, morphology and trap distribution were also investigated. Results show that IPCs with a 15% rubber phase content (IPC15) can achieve the simultaneous improvement of elongation at break and breakdown strength compared with isotactic PP, which can be attributed to the special crystalline structure. According to the results of differential scanning calorimetry (DSC) and FTIR, it is proposed that the lamella thickness of IPC15 is maximal and some ethylene segments exist in PP crystals of IPC15 as crystalline structure defects, which is responsible for this enhanced breakdown strength. The morphology results reveal that rubber microspheres are found to coexist with spherulites, which can promote the relative sliding among lamellas under external force and further results in the increase in the elongation at break.

## 1. Introduction

Polypropylene (PP)-based thermoplastic insulation has attracted tremendous interest because of its high temperature stability and excellent recyclability in the past. Compared with traditional cross-linked polyethylene (XLPE) insulation, PP possesses advantages of not only an increased operating temperature but also facile manufacturing processing with reduced costs [1]. The production efficiency of cables insulated by thermoplastic insulation can also be boosted by the absence of cross-linking and degassing [2]. However, conventional PP cannot be used directly as power cable insulation duo to its high modulus, low flexibility and poor aging resistance [3,4]. Hence, in recent years, many scholars have been committed to optimizing PP as cable insulation.

Hosier et al. investigated the effect of ethylene content ranging from 2 to 40% on the mechanical and electrical properties of the ethylene–propylene random copolymer [5,6]. Results showed that the melting point and tensile modulus of the copolymer both decreased with the increase in ethylene content, indicating that ethylene can enhance the flexibility of PP copolymers. However, the elongation at break of all samples was not high enough to meet the processing conditions of cable insulation. The breakdown strength of isothermally crystallized samples also declined with an increased ethylene content. Du et al. evaluated the electrical and mechanical properties of isotactic PP blended with a polyolefin elastomer (POE) and propylene-based elastomer (PBE) [7]. It was pointed out that the elongation at break of both PP/PBE and PP/POE samples significantly increased with an increased elastomer content, however, the trap depth and breakdown strength of both PP/PBE and PP/POE samples tended to be reduced. According to these studies, it can be seen that a random copolymerizing with ethylene or blending with elastomer can both enhance the mechanical property of PP, but the degradation of electrical property seems inevitable. Zhou et al. reported a ternary blending system composed by PP/thermoplastic polyolefin (TPO)/MgO which can synergistically improve the electrical and mechanical properties of PP [8]. However, from the perspective of engineering applications, achieving a uniform dispersion of nanoparticles during cable extrusion is a problem. Christian Müller et al. synthesized an ethylene-propylene copolymer, which showed good compatibility with low-density polyethylene (LDPE). They also found that the blend of polyethylene (PE)–PP copolymers and LDPE exhibited excellent melt creep resistance and dielectric properties at operating temperatures [9,10]. It can be seen that a multisystem with good compatibility can synergistically improve the mechanical and electrical properties of the polymer, but the high cost caused by the complex production process is also an issue that has to be considered. Therefore, it is important to seek directly synthesized polymers with excellent electrical and mechanical properties.

Impact polypropylene copolymers (IPCs) are a typical multiphase in-reactor alloy, which are composed of isotactic PP (iPP), amorphous ethylene–propylene rubber and ethylene–propylene block copolymers with different sequence lengths as a nature compatibilizer [11,12]. The complex processing steps can be avoided and the compatible multiphase structure can be easily achieved through the method of direct synthesis in the reactor. Exploring the possibility of using IPCs as cable insulation will be of great significance to the cable industry. At present, some studies have reported the improvement in the toughness of IPCs than iPP [13,14], however, there is little research on the electrical performance of IPCs. Meanwhile, the synthesis process and elastomer content of the material are not clear in the relevant research on the breakdown performance of flexible PP, and it is difficult to provide references for further optimizing of IPCs [15]. In this paper, the tensile and breakdown properties of IPCs with different ethylene–propylene rubber phase contents were evaluated, moreover, the morphology, crystallinity and trap distribution were also investigated to analyze the influence mechanism of the morphology on electrical properties. The aim of this work is to explore the dependence of electrical and mechanical properties on the microstructure of IPC, which can provide a reference for the optimization of modified PP for cable applications.

## 2. Materials and Methods

### 2.1. Sample Preparation

IPCs with ethylene–propylene rubber phase of 15 and 30% were synthesized using conventional Ziegler–Natta catalyst by a two-step polymerization. An iPP sample and an XLPE for a 35 kV cable application were also prepared for comparison. All samples in this paper were provided by SINOPEC Beijing Yanshan Company (Beijing, China). IPCs with rubber phases of 15 and 30% were named IPC15 and IPC30, respectively, in this paper. IPC and iPP samples were prepared by compression molding. First, the polymer pellets were heated in a mold for 5 min at 200 °C, then hot-pressed under a pressure of 5, 10 and 15 MPa for 5 min, respectively. When switching pressure, the pressure is reduced to 0 MPa first and then rose to the specified pressure. Afterwards, the mold was moved to the other layer of the compression machine with a water-cooling system and was pressed under a pressure of 5 MPa until completely cooling down. The cooling rate was set at around 5 °C/min. Samples about 100 μm in thickness were used for the breakdown strength, morphology and structure measurements. Samples about 1 mm in thickness were used for mechanical property measurements. The preheating temperature and molding temperature for the XLPE samples were set as 120 and 180 °C. The molding pressures of XLPE were the same as iPP and IPC samples, and the pressure was removed for 1 min to avoid bubbles. The main degassing process of XLPE was conducted in the vacuum oven at 70 °C for 24 h. In order to ensure the consistency of the sample, the iPP and IPC samples were also processed in the same way.

### 2.2. Characterization

For the observation of the crystallizing process, polarized optical microscopy (POM) (Olympus bx51-P, Tokyo, Japan) with a Linkam THMS 600 (Olympus, Tokyo, Japan) hot stage was used. The sample was compressed to about 50 μm in thickness in order to observe the spherulite structure more clearly. During the observation process, the hot stage was heated up to 200 °C at a heating rate of 10 °C/min and was cooled down to 80 °C at a cooling rate of 5 °C/min after keeping at 200 °C for 5 min. The spherical morphology was recorded during the cooling process. Moreover, the fracture surface morphology after xylene etching was observed by a scanning electron microscope (SEM) (Keyence VE-9800S, Osaka, Japan).

The melting and crystallizing behavior of samples were investigated by a differential scanning calorimetry (DSC) 822e (METTLER TOLEDO, Zurich, Switzerland) instrument under a nitrogen atmosphere with a flow rate of 40 mL/min. The samples were heated from 30 up to 200 °C at a heating rate of 10 °C/min and were cooled down to 80 °C at a cooling rate of 5 °C/min after keeping at 200 °C for 5 min. The sample weight was 5-6 mg and the crucibles were made of aluminum.

The FTIR spectra were obtained by a Nicolet iN10 (Thermo Scientific, Waltham, MA, US) spectrometer in attenuated total reflectance mode with wave number varying from 4000 to 400 cm^-1^. The condition of ethylene segments can be reflected in the infrared spectrum.

Surface potential decay (SPD) experiments were performed to study the trap distribution and inner charge transport characteristics. More detail of the test system has been described in our previous work [16]. In the measurements, the needle electrode and mesh grid were applied with constant direct–current voltage at −13 and −3 kV, respectively. The charging times were set as 3 min and the measurements were carried out for 2 h.

An alternating–current (AC) breakdown test was conducted on films about 100 μm in thickness in transformer oil at room temperature by a computer-controlled voltage breakdown testing machine (HJC–100 kV, Huayang Instruments, Fosha, Guangdong, China). An increasing AC electric field was applied to the samples at a rate of 1 kV/s until breakdown. A ball electrode system with a diameter of 25 mm was used and 10 points were tested for each sample. In the process of researching the results of the breakdown test, a two-parameter Weibull distribution is often used to fit the relationship between the cumulative probability of failure and breakdown strength [17]. The breakdown strength at the cumulative probability of 63.2% is called the Weibull breakdown strength α. There is also parameter β representing the inverse of data scatter.

The tensile property was obtained by CMT-4503 mechanical tensile machine (Meitesi Industry System, Shenzhen, China). Dumbbell shaped samples for the tensile test were cut from sheet specimen with a thickness of 1 mm. The neck length of samples was 25 mm and the cross section of neck was 4 mm × 1 mm. The stretching rate was set at 100 mm/min and the gauge length was 20 mm. At least 6 repeated experiments were conducted for each sample.

## 3. Results

### 3.1. Enhanced AC Breakdown Strength and Elongation at Break of IPC

**Figure 1:**
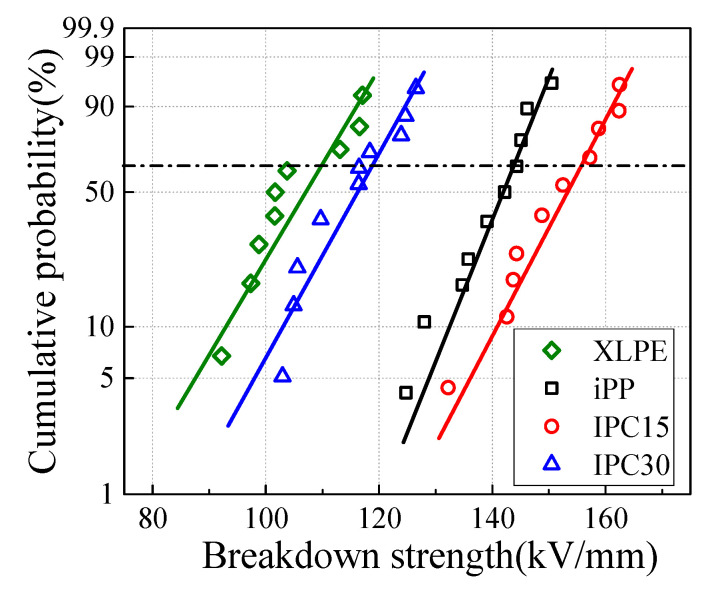
Weibull plots of AC breakdown strength.

The Weibull plots and the Weibull parameters are given in Figure 1 and Table 1, respectively. The β of all samples is larger than 10, suggesting good data consistency. The AC breakdown strengths of the iPP and IPC samples are higher than that of XLPE, indicating that iPP and IPCs have the potential to increase the cable operating voltage and reduce insulation thickness. The sequence of α for iPP and IPCs from high to low are: IPC15, iPP and IPC30, which indicates that the IPC can achieve a higher breakdown strength than iPP when the rubber phase is reasonable. Compared with iPP, the breakdown strength of IPC15 enhances from 143.39 to 154.88 kV/mm, which is converse to the declined breakdown strength blend with elastomer in some references [7,18,19]. The breakdown strength of IPC30 shows a significant decline compared to IPC15, with a falling ratio about 24.0%, which indicates that the presence of an excessive rubber phase can result in the decrease in breakdown strength.

**Figure 2:**
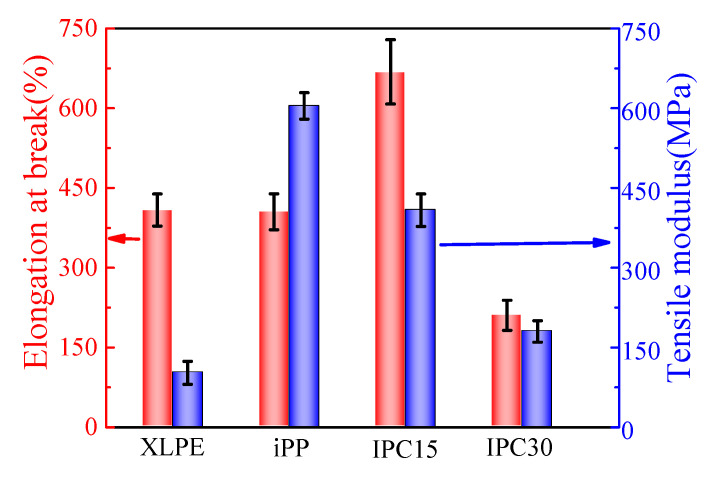
Elongation at break and tensile modulus of all samples.

The tensile modulus and elongation at break of all samples are shown in Figure 2. It can be seen that the tensile modulus of IPCs decreases with the increase in rubber phase content, which indicates that the high moduli of iPP are significantly optimized. The elongation at break of IPC15 is obviously higher than that of iPP. Compared with iPP, the tensile modulus of IPC15 decreases by about 32.4% while the elongation at break increases by about 64.9%. However, a significant drop of elongation at break is observed in the result of IPC30, which is only a half of XLPE.

According to the results of the breakdown strength and tensile experiment, it can be seen that the simultaneous improvement of mechanical and electrical properties can be achieved through an impact copolymerization when the rubber phase content is proper, which is better than simple blending modifications. The mechanism of achieving the synergistic improvement of electrical and mechanical properties by impact copolymerization will be discussed in detail below.

### 3.2. Differences of Crystalline Structure Between IPC and iPP

**Figure 3:**
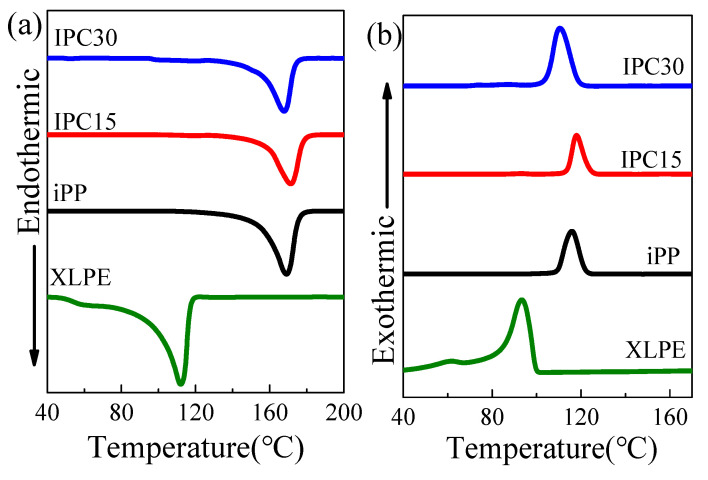
Melting (**a**) and crystallization (**b**) curves of the samples.

The DSC curves of all samples are exhibited in Figure 3. The melting temperature *T*_m_, crystallization temperature *T*_c_, melting enthalpies Δ*H*_m_ and crystallinity *X*_c_ are listed in Table 2. It can be seen that the *T*_m_ of iPP and IPCs are higher than XLPE, indicating that a polypropylene insulating cable can withstand higher temperatures in the actual operating process. This phenomenon has important implications for improving the transmission capacity of power cables. Moreover, the *T*_m_ of the iPP and IPCs exhibits a slight change, and similar behaviors are found in the crystallization processes as well, as shown in Figure 3b. The *T*_c_ of IPCs and iPP are both within the crystallization temperature range of PP segments, which revealed that the crystalline part of the samples are still mainly PP segments. An interesting phenomenon is that the *X*_c_ of IPC15 is found to be slightly less than that of iPP. In other words, the crystallinity of the part except the rubber phase in IPC15 is even higher than that of iPP. However, the *X*_c_ of IPC30 is less than that of iPP and IPC15, indicating that an excessive rubber phase may be harmful to the crystallinity. The lamellar thickness of the samples can be calculated on the basis of Thomson–Gibbs equation, which is expressed as follows [20].

(1) $$L=\frac{2\sigma_{e}T_{m0}}{\Delta H_{m}\left(T_{m0}-T_{m}\right)}$$

where *L* is the average lamellar thickness, nm, *T*_m0_ is the equilibrium melting temperature of an infinite crystal, K, *σ*_e_ is the surface free energy per unit area of the basal plane, Jm^−2^, Δ*H*_m_ is the melting enthalpy per unit volume, Jm^−3^. These parameters can be found in reference [20]. In this paper, the parameters are selected as: *T*_m0_ = 460.7 K, *σ*_e_ = 10^−1^ Jm^−2^ and Δ*H*_m_ = 1.54 × 108 Jm^−3^. The calculated average lamellar thicknesses of iPP, IPC15 and IPC30 are 31.99, 36.04 and 30.37 nm, respectively. It can be seen that the lamellar thicknesses of IPC15 is larger than the other two samples and the lamellar thicknesses of IPC30 is the smallest, which indicates that proper a rubber phase can increase the lamella thickness.

**Figure 4:**
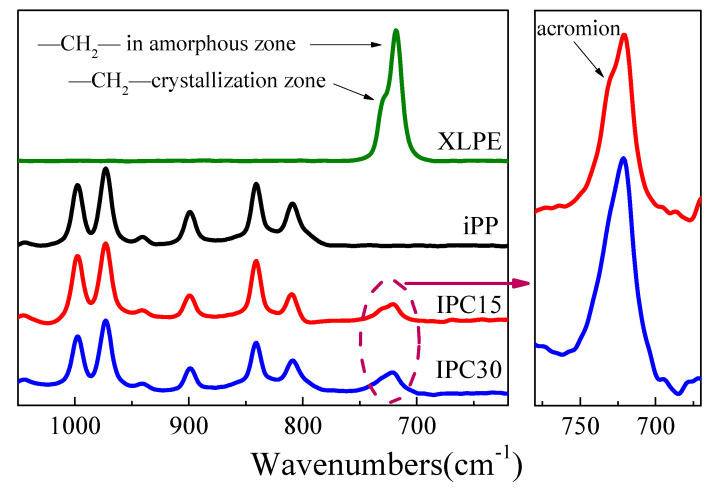
FTIR curves of the polypropylene (PP) and impact PP copolymers (IPCs).

The FTIR of PP and IPCs has been used to investigate the condition of ethylene segments in IPCs. The testing results of all samples are exhibited in Figure 4. It can be seen that strong bands appear at 730 and 720 cm^−1^ in the curve of XLPE while no obvious bands can be observed at the same position in the curve of iPP, indicating that these two bands can be seen as the characteristic bands of ethylene segments in IPCs.

The reported bands at 730 and 720 cm^−1^ of the infrared spectrum of PE correspond to the rocking vibration of –(CH_2_)n– (n ≥ 5) in the crystalline region and the amorphous region, respectively [21]. Based on this conclusion, it is suggested that the ethylene segments mainly exist in the amorphous zone of IPCs. Specially, a shoulder band can be found at 730 cm^−1^ in the curve of IPC15, which revealed that some ethylene segments exist in the crystalline region. However, according to the results of DSC in Figure 3, only the melting and crystalline bands of PP crystals can be found and no melting and crystalline bands related to PE crystal can be seen, which revealed that these ethylene segments existing in the crystalline region are not from a PE crystal. A possible reason for this interesting phenomenon is that the crystalline ethylene segments of IPC15 is from the crystal composed by a long PP molecular chain with a few ethylene segments. In other words, there are some PP crystals that contain ethylene segments as crystalline structure defects in IPC15. In the spectrum band of IPC30, no shoulder band can be found, which emphasizes that it is difficult to form such a PP crystalline structure containing ethylene segments when the rubber phase content is excessive.

### 3.3. Morphology of PP and IPCs 

**Figure 5:**
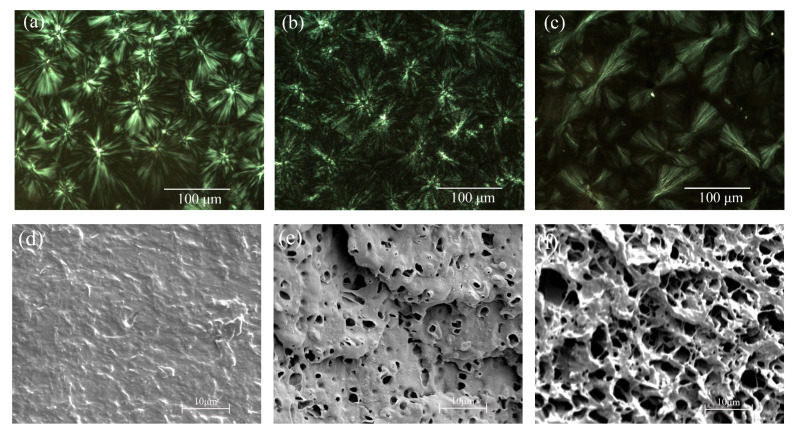
Crystalline morphology and fractured surfaces structure of iPP (**a,d**), IPC15 (**b,e**) and IPC30 (**c,f**).

Figure 5a–c show the spherulite morphology of PP and IPCs observed by POM after the nonisothermal crystallization process at a cooling rate of 5 °C/min. The spherulites of iPP are well-defined, and the spherulites impinge on each other, forming many apparent boundaries. This is the typical spherulite morphology of PP with high isotacticity. The spherulite structure of IPCs is found significantly deteriorated, which is considered to be the result of the rubber phase dispersing in the spherulites and interspherulitic regions [22,23]. Moreover, the definition of Maltese-crosses is found clearly to decrease the image of IPC30, indicating the increased irregularity of the crystalline structure and the decrease in crystallinity. Figure 5d–f show the image of fractured surfaces of iPP and IPCs etched by xylene, which can be used to investigate the distribution of ethylene–propylene rubber phase. It can be seen that IPCs exhibit a characteristic ‘‘sea–island’’ structure, where the “islands” represent the rubber phase and the “sea” is the PP matrix. IPCs and iPP present very different rubber phase sizes. Compared with IPC15, the rubber phase size of IPC30 significantly increases and the distribution of rubber phase becomes wider, which corresponds to the shaded area in Figure 5c.

### 3.4. Trap distribution of iPP and IPC

**Figure 6:**
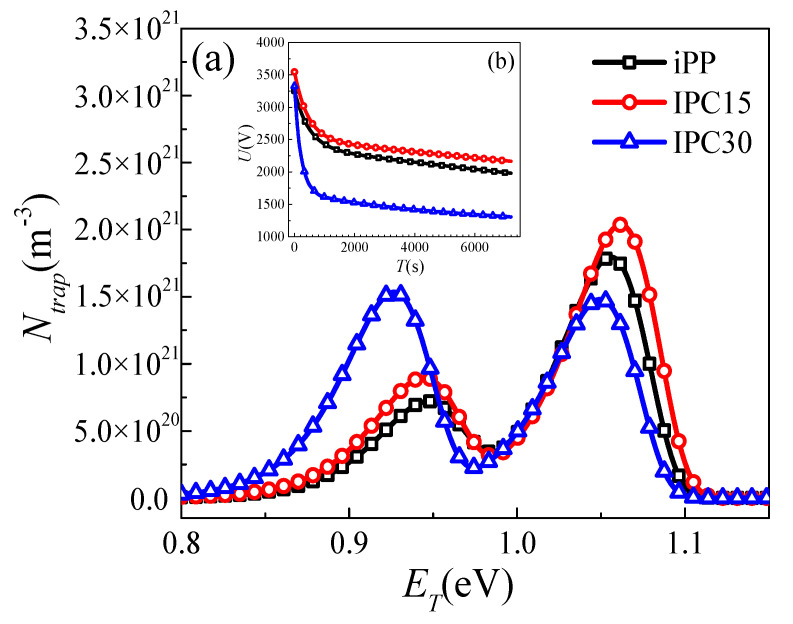
Trap distribution (**a**) and primitive curve (**b**) of surface potential decay (SPD).

In order to understand the relation between the microstructure and breakdown strength of IPCs, the trap distribution was calculated based on the method in our previous work [24]. Figure 6 shows the primitive curves acquired by SPD and the trap distribution curves. It can be seen that the SPD curves show a monotonic decrease with an increasing decay time of PP and IPCs. The initial surface potentials of three samples are closed, which indicates a good consistency and comparable results of these experiments. With the increase in the rubber phase content, the energy level (*E_T_*) and trap density (*N_trap_*) of shallow traps and deep traps show different variations. The *E_T_* of shallow traps gradually decrease and the *N_trap_* of shallow traps sharply increase. Meanwhile, the *E_T_* and *N_trap_* of deep traps show a trend of rising first and then falling. The detailed data of *E_T_* and *N_trap_* are listed in Table 3. Compared with iPP, the *E_T_* and *N_trap_* of deep traps change from 1.055 eV and 1.79 × 10^21^ m^−3^ to 1.063 eV and 2.04 × 10^21^ m^−3^ when those of shallow traps change from 0.949 eV and 7.43 × 10^20^ m^−3^ to 0.942 eV and 9.36 × 10^20^ m^−3^ in IPC15.

## 4. Discussion

### 4.1. Schematic Diagram of the Spherulite Structure Evolution Affected by the Rubber Phase

**Figure 7:**
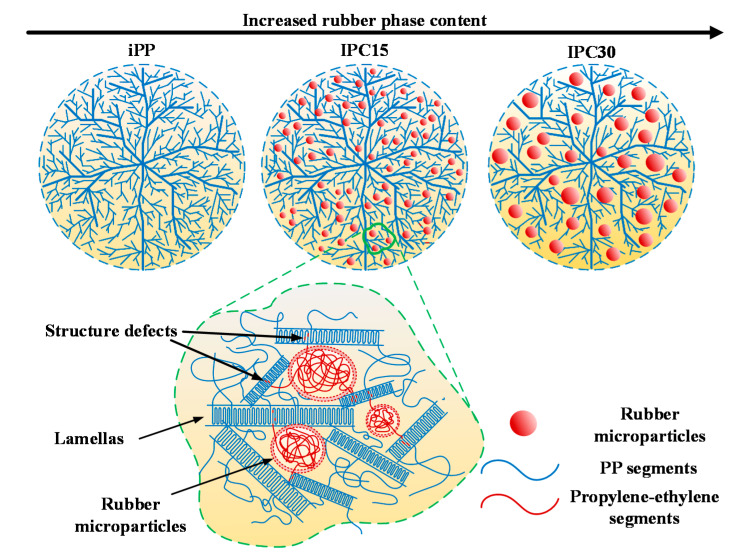
Schematic diagram of structure evolution affected by rubber phase content.

Based on the morphology observed by a microscopic examination and crystalline structure analysis in the previous chapter, the schematic diagram of the spherulite structure containing a rubber phase is presented, as shown in Figure 7. IPP shows perfect spherulites with no amorphous microsphere, which corresponds to the result in Figure 5a. In IPC15, many amorphous microspheres distributed in the spherulites are found. Due to the huge size difference between spherulites and the rubber phase, a partially enlarged view of IPC15 is provided to exhibit the distribution of ethylene–propylene chains. It can be seen that the rubber phase existed among PP lamellas. According to the results of DSC and FTIR, some PP crystals containing ethylene segments can be found in IPC15. The PP crystal containing ethylene segments can be regarded as a defective crystal, which may impact the trap distribution of materials and further affect the breakdown strength. In IPC30, the size of the rubber phase is significantly increased and the number of lamella decline obviously, which is consistent with the results of SEM and the sharply decreased crystallinity of IPC30. Furthermore, the ethylene molecular chain segments almost all exist in amorphous zones.

### 4.2. Role of the Rubber Phase on the Mechanical Properties

According to the results of tensile experiment, the elongation at break shows a maximum value in IPC15 and the tensile modulus of IPC15 is also significantly lower than that of iPP. The high elongation at break of IPC15 can be explained from the following two aspects. On the one hand, the apparent boundaries of iPP are the stress concentration area, in which structure defects are easy to be produced under external force. The degraded spherulites boundaries of IPC15 can facilitate the dispersion of stress, and further improve the elongation at break [25]. On the other hand, based on previous research in this paper, many rubber microspheres can be found in the crystalline region in IPC15, as shown in Figure 5 and Figure 7. The existing amorphous microspheres can make the relative sliding among lamellas easier under external force, which can result in the increase in elongation at break [26]. Moreover, the elongation at break of IPC30 is sharply lower than that of iPP and IPC15, which indicates that an excessive rubber phase cannot achieve a good toughening effect. 

### 4.3. Relation Between the Microstructure and Trap Distribution of IPC

**Figure 8:**
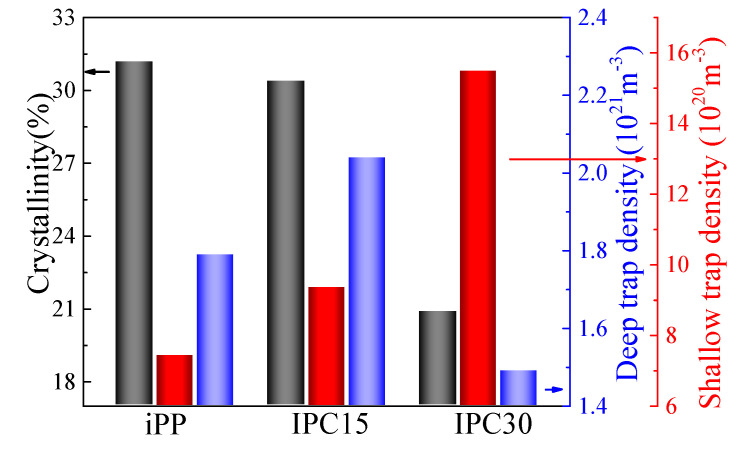
The crystallinity and trap density of iPP and IPCs.

The crystallinity and *N_trap_* of iPP and IPCs are exhibited in Figure 8. It can be seen that IPC30 exhibits the highest shallow trap density and lowest deep trap density. In semicrystalline polymers, the deep traps are often thought to exist in the crystalline regions while the shallow traps are more in the amorphous regions with a low-density [27]. According to the changes in crystallinity and trap density, it is suggested that the lowest crystallinity is responsible for the high shallow trap density and low deep trap density of IPC30. In addition, both the shallow and deep trap density of IPC15 are higher than that of iPP while the crystallinity of IPC15 is a little less than iPP, which revealed that there are other structural changes that can cause the change of deep traps in addition to the crystallinity. Based on previous research, crystalline structure defects are always regarded as the origin of deep trap [28]. In the previous chapter, the ethylene segments in the PP crystal could only be found in IPC15, which may be responsible for the increase in the deep trap density when the crystallinity of IPC15 was found to be slightly lower than that of iPP.

### 4.4. Mechanism of the Enhanced Breakdown Strength of IPC

**Figure 9:**
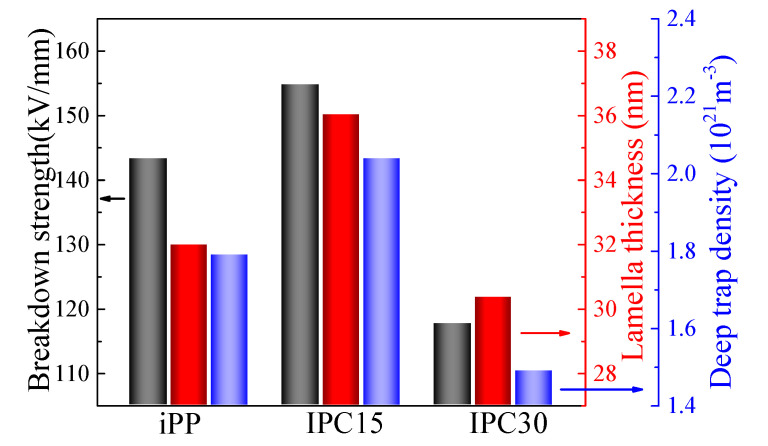
The lamella thickness, deep trap density and breakdown strength of iPP and IPCs.

The breakdown of polymers is a complex process, which is impacted by many factors, such as the crystalline structure, crystallinity, trap distribution and so on. Based on previous research, the dependence of the breakdown strength on the lamella thickness and deep trap density are discussed. The breakdown strength, lamella thickness and deep trap density of iPP and IPCs are exhibited in Figure 9. It can be seen that the greater the lamella thickness and the density of deep traps, the higher the breakdown strength. The dependence of the breakdown strength and lamella thickness has been extensively studied [17,29]. At present, the mainstream view holds that breakdown strength is positively correlated with the lamella thickness. The larger the lamellae thickness, the higher the breakdown strength. The results of DSC show that the sequence of lamella thickness of PP and IPCs from high to low are: IPC15, iPP, IPC30, which is one of the reasons for the highest breakdown strength of IPC15.

**Figure 10:**
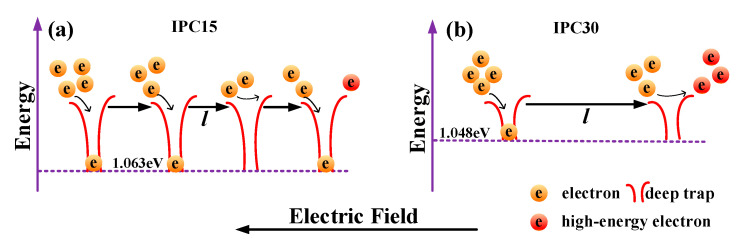
Schematic diagram of deep trap modulated carrier transport in IPC15 (**a**) and IPC30 (**b**).

Trap modulating breakdown may be another factor that affects the breakdown strength. Electrons can be accelerated into high-energy electrons under a strong electric field and further destroy molecular chains, which is one of the main causes of breakdown. As the trap energy level increases, the residence time of electrons in the trap will be significantly prolonged, which suggests that the effect of deep traps on the electron transport process is significantly stronger than that of shallow traps. Therefore, the role of deep traps will be mainly explored. A schematic diagram of the deep trap modulating breakdown strength is shown in Figure 10. In IPC15, both the number of encountered traps during the electron migrating process and the probability of being trapped increase. The residence times of trapped electrons are relatively long due to a higher trap level. Only few electrons can be accelerated through enough distance under an electric field to become high-energy electrons as shown in Figure 10a, hence the breakdown strengths of IPCs are excellent. In IPC30, the probability of being trapped sharply decreases and the probability of detrapping increases. More high-energy electrons can be generated than IPC15 after accelerating them under an electric field at same distance, which can further lead to the decreased breakdown strength of IPC30.

## 5. Conclusions

In this paper, the morphology, trap distribution, electrical properties and mechanical properties of PP and IPCs with different ethylene–propylene rubber phase content were investigated. The main conclusions are exhibited below:

(1) Impact copolymerization is an effective way to optimize the electrical and mechanical properties of iPP simultaneously. Compared with iPP, the breakdown strength and elongation at break of IPC15 increase about 8.0 and 64.9%, respectively. However, a sharp decrease in the breakdown strength and elongation at break can be found with an excessive rubber phase content.
(2) The enhanced breakdown strength of IPC15 is attributed to the impact of lamella thickness and crystalline structure defects. The lamella thickness of IPC15 is larger than iPP and IPCs, which leads to an elevated breakdown strength. It is also suggested that the crystalline structure defects caused by ethylene segments in the PP crystal are responsible for the increase in the deep trap density and level of IPC15, which can further lead to an increased breakdown strength. The high elongation at break of IPC15 is influenced by the rubber microspheres among the lamellas. The elongation at break can be increased by rubber microspheres through enhancing the relative sliding among lamellas under external force and reducing the stress concentration.
(3) The sharply decreased breakdown strength of IPC30 is considered to be caused by the expansion of amorphous regions. An increased rubber phase content leads to a significant decrease in crystallinity, which further results in the decrease in the deep trap level and density. It was found that the lamella thickness of IPC30 is the thinnest. The breakdown strength sharply declined due to the decreased lamella thickness and deep trap.

## Figures and Tables

**Table 1:** Weibull parameters of AC breakdown strength.

Sample Code	Weibull Parameters	
	α	β
XLPE	108.6 ± 8.4	13.5
iPP	143.4 ± 7.8	27.5
IPC15	154.9 ± 9.4	22.2
IPC30	117.8 ± 8.3	15.8

**Table 2:** Main differential scanning calorimetry (DSC) data and lamella thickness of the samples.

Sample Code	*T*_m_(°C)	*T*_c_(°C)	Δ*H*_m_(J/g)	*X*_c_(%)	*L*_(nm)_
XLPE	112.1	93.3	79.87	27.8	/
iPP	169.0	116.0	65.13	31.2	31.99
IPC15	171.1	118.1	63.47	30.4	36.04
IPC30	168.0	110.6	43.57	20.9	30.37

**Table 3:** Trap distribution parameters.

Sample Code	*E*_T_ (eV)		*N*_trap_ (m^−3^)	
	Shallow	Deep	Shallow	Deep
iPP	0.949	1.055	7.43 × 10^20^	1.79 × 10^21^
IPC15	0.942	1.063	9.36 × 10^20^	2.04 × 10^21^
IPC30	0.928	1.048	1.55 × 10^21^	1.49 × 10^21^
